# Prestin as a potential biomarker in peripheral blood: differential expression across hearing loss subtypes and acute inner ear injury

**DOI:** 10.1016/j.clinsp.2026.101143

**Published:** 2026-10-15

**Authors:** Xuan Sun, Dian Wang, Yahan Zhao, Zhenghan Zhu, Lingxiang Hu, Yi Li

**Affiliations:** a Beijing Tongren Hospital, Capital Medical University, China; b The Second Affiliated Hospital of Nanjing Medical University, China; c Shanghai 9th People’s Hospital Affiliated to Shanghai Jiaotong University School of Medicine, China

**Keywords:** Prestin, Biomarker, Conductive hearing loss, Sudden sensorineural hearing loss, Hearing impairment

## Abstract

**Objective:**

This exploratory study investigated peripheral blood Prestin levels across different hearing loss subtypes to evaluate its potential as a biomarker for inner ear injury.

**Methods:**

Plasma Prestin levels were measured via ELISA in 100 age-matched participants, comprising a healthy control group (HCG, n = 23) and patients with conductive (CHLG, n = 29) and unilateral sudden sensorineural hearing loss (USSNHLG, n = 48). Associations with hearing loss subtypes, initial audiogram configurations, and treatment outcomes were analyzed.

**Results:**

Overall, Prestin levels varied significantly across the three groups (P < 0.001). Following Dunn’s test with Bonferroni correction, Prestin levels in the USSNHLG were significantly higher than those in the HCG (P = 0.019), whereas levels in the CHLG were significantly lower than those in the HCG (P = 0.014). Within the USSNHLG, no statistically significant differences in Prestin levels were observed among patients with different therapeutic outcomes (P = 0.102).

**Conclusions:**

The present findings confirm that acute inner ear injury (USSNHL) is associated with significantly elevated peripheral blood Prestin levels, whereas persistently reduced acoustic input (CHL) is associated with decreased levels. However, current data do not support baseline Prestin as a prognostic indicator for USSNHL. Future multicenter studies with strict clinical stratification are required to fully establish its clinical utility.

## Highlights

• This is the first human Prestin study targeting various types of hearing loss.
• It provides Prestin levels of normal people and patients with hearing loss.
• It evaluates the potential of Prestin as a pathological biomarker for the ear.
• It explores the implications of Prestin in different pathological conditions.

## Introduction

Prestin is a specific motor protein located on the lateral membrane of Outer Hair Cells (OHCs). Exhibiting high tissue specificity, it plays a critical role in the active amplification process of OHC electromotility.^1^ Animal studies have demonstrated that the concentration of Prestin in peripheral blood may serve as a reliable biomarker for noise-induced or ototoxic damage to the auditory system.^2^, ^3^, ^4^, ^5^ Given its capacity to reflect inner ear injury, recent research has proposed that peripheral blood Prestin levels in patients with Sudden Sensorineural Hearing Loss (SSNHL) can indicate the severity of inner ear lesions. This offers significant insights into the pathological mechanisms, disease progression, and clinical outcomes of SSNHL and other inner ear disorders.^6^, ^7^

However, the interpretation of fluctuating peripheral blood Prestin levels under pathological conditions is complicated by numerous theoretical factors. These include the dynamic equilibrium of OHC lateral membrane protein turnover, the extent of OHC damage and subsequent repair, and the permeability of the Blood-Labyrinth Barrier (BLB). Under pathological states, the precise contributions of these variables to peripheral blood Prestin levels remain elusive. Therefore, as a preliminary exploratory study, this research aims to investigate the association between peripheral blood Prestin levels and various types of hearing loss. Furthermore, it seeks to establish reference values for Prestin levels under healthy physiological conditions, as well as in cases of Conductive Hearing Loss (CHL) and acute pathologies such as SSNHL.

## Materials and methods

### Study subjects and grouping criteria

(1) Healthy Control Group (HCG): To establish baseline data, this study enrolled 23 healthy adults with normal hearing as controls.
(2) Conductive Hearing Loss Group (CHLG): Although patients with conductive hearing loss lack inner ear pathology, the reduced acoustic stimulation to the inner ear may also induce alterations in peripheral blood Prestin levels. To assess Prestin levels under such conditions, the authors included 29 patients with CHL scheduled for otologic surgery at Beijing Tongren Hospital, between September 2024 and December 2024. All patients had a disease duration exceeding three months. Diagnostic Criteria for CHL: Bone conduction Pure-Tone Average (PTA) thresholds (250–4000 Hz) ≤ 20 dB HL in both ears.
(3) Unilateral Sudden Sensorineural Hearing Loss Group (USSNHLG): To evaluate peripheral blood Prestin levels under acute pathological conditions, 48 patients with USSNHL admitted to Beijing Tongren Hospital between November 2024 and December 2024 were enrolled. Peripheral blood samples were collected during the initial acute phase for Prestin measurement. Diagnostic Criteria: Idiopathic sensorineural hearing loss occurring within a 72-hour window, characterized by a threshold elevation of ≥30 dB HL across at least three consecutive frequencies.^8^

### Treatment protocol and efficacy evaluation for USSNHL patients

All enrolled patients with USSNHL were classified into clinical subtypes based on the PTA thresholds and audiogram configurations of the affected ear during the initial onset phase. Audiogram configurations were categorized as upsloping, downsloping, flat, or profound. Tailored treatment regimens were initiated in the otolaryngology emergency department of the studied institution within 14-days of symptom onset, strictly adhering to the Chinese clinical guideline “Diagnosis and Treatment Guideline for Sudden Deafness (2015)”.^9^

Upsloping & Downsloping Subtypes: Combined therapy with glucocorticoids and Ginkgo biloba extract.

Flat & Profound Subtypes: Triple therapy with glucocorticoids, Ginkgo biloba extract, and baclofen.

As peripheral blood Prestin levels in these patients were measured prior to treatment initiation, the specific therapeutic regimens did not confound the primary conclusions of this study. Treatment was discontinued early if the hearing thresholds of the affected ear returned to baseline levels within the 14-day intervention period. Fourteen days post-treatment, PTA thresholds were re-evaluated, and therapeutic outcomes were graded according to Siegel’s criteria.^10^ For statistical analysis, “partial recovery” and “slight recovery” in Siegel’s criteria were merged and defined as “significant improvement” in this study.

### Blood sample collection and preservation

Peripheral blood samples were collected from the patient groups described above using sodium citrate anticoagulant tubes (pre-loaded with 0.2 mL sodium citrate). Specifically, 1.8 mL of venous blood was drawn from the antecubital vein, gently inverted for mixing, and centrifuged within one hour of collection. Centrifugation was performed at 3000 rpm for 20 minutes to isolate the plasma. The separated plasma was then aliquoted and stored at 4 °C for a maximum of one week, or at −20 °C for no longer than one month.

### Prestin concentration measurement via ELISA

Prestin concentrations were quantified using a commercial ELISA kit (Human Prestin [SLC26A5] ELISA Kit #EK11371, Signalway Antibody) with undiluted plasma samples. All procedures strictly adhered to the manufacturer’s protocol. Following color development, absorbance was measured at 450 nm and 492 nm. The final Optical Density (OD) was calculated by subtracting the 492 nm background reading from the 450 nm reading. Prestin concentrations were derived from a standard curve generated through duplicate measurements of calibrators, whereas patient samples were measured in triplicate.

### Statistical methods

All statistical analyses were performed using SPSS Statistics 29 and GraphPad Prism 10. Continuous variables with a normal distribution are presented as mean ± Standard Deviation (SD), while non-normally distributed variables are expressed as Median and Interquartile Range (M, IQR).

Overall differences among multiple groups with non-normally distributed data were assessed using the Kruskal-Wallis *H*-test (significance level α=0.05). Post hoc comparisons were conducted using Dunn’s test with Bonferroni correction; since only two biologically meaningful pairwise comparisons were performed, the adjusted significance level was set at α’=0.025. Differences between two independent groups with non-normally distributed data were analyzed using the non-parametric Mann-Whitney *U test* (α=0.05). A two-tailed statistical hypothesis testing approach with a 95% Confidence Interval (95% CI) was adopted, and statistical significance was defined as p < 0.05 for all comparisons.

The reporting of this study conforms to the Strengthening the Reporting of Observational Studies in Epidemiology (STROBE) guidelines.

## Research results

### Baseline characteristics of the study groups

Table 1 summarizes the baseline clinical characteristics of the enrolled groups. The median ages of the HCG, CHLG, and USSNHLG were 51.00, 48.00, and 48.50 years, respectively. The Kruskal-Wallis *H*-test revealed no statistically significant differences in age among the three groups (*H* = 0.76, df=2, p = 0.684). Similarly, Fisher’s exact test was used for sex distribution analysis due to small expected frequencies in some cells, and no significant difference was found among the groups (χ^2^=1.40, df=2, p = 0.497). These findings indicate excellent baseline comparability across the three groups regarding age and sex.

**Table 1:** Baseline characteristics of the three groups.

**Variables**	**HCG** **(n=23)**	**CHLG** **(n=29)**	**USSNHLG** **(n=48)**
Age (M, IQR)	51 (29, 59)	48 (33.5, 57.5)	49.5 (38, 61)
Sex			
Male/Female	14/9	17/12	23/25
Pure Tone Average (dB)			
AC (250‒8000 Hz)	3.17±3.46	35.57±10.79	46.46±17.72
BC (250‒4000 Hz)	‒	12.32±4.78	‒

Pure-Tone Average (PTA): As the HCG and USSNHLG exhibited no air-bone gaps, their bone conduction thresholds were equivalent to their air conduction thresholds and are therefore not reported separately.

Additionally, the table provides the Pure-Tone Average (PTA) thresholds of both ears for clinical reference. Air Conduction (AC) thresholds were calculated as the average across six pure-tone frequencies (250–8000 Hz), whereas Bone Conduction (BC) thresholds were averaged across five frequencies (250–4000 Hz). Notably, because no air-bone gaps were observed in the HCG and USSNHLG, their hearing levels are represented exclusively by AC thresholds (Table 1).

### Peripheral blood Prestin levels across the three groups

Fig. 1 displays the peripheral blood Prestin levels across the three groups. The HCG exhibited low dispersion in Prestin levels, with a median concentration of 127.66 pg/mL (Interquartile Range [IQR]: 99.86–144.18 pg/mL), establishing a reliable baseline. The CHLG showed the lowest Prestin levels (median: 79.75 pg/mL, IQR: 44.46–101.71 pg/mL) with minimal dispersion. In contrast, the USSNHLG demonstrated markedly elevated Prestin levels with high dispersion (median: 234.37 pg/mL, IQR: 112.51–426.76 pg/mL). The Kruskal-Wallis *H*-test revealed a highly significant overall difference among the three groups (*H* = 23.15, df=2, p < 0.001). Post hoc analysis using Dunn’s test with Bonferroni correction demonstrated that Prestin levels in the HCG were significantly higher than those in the CHLG (*Z* = 2.46, p = 0.014) and significantly lower than those in the USSNHLG (*Z*=−2.34, p = 0.019). The pairwise comparison between CHLG and USSNHLG was not performed as it was not biologically relevant to the primary research questions of this study.

**Fig. 1:**
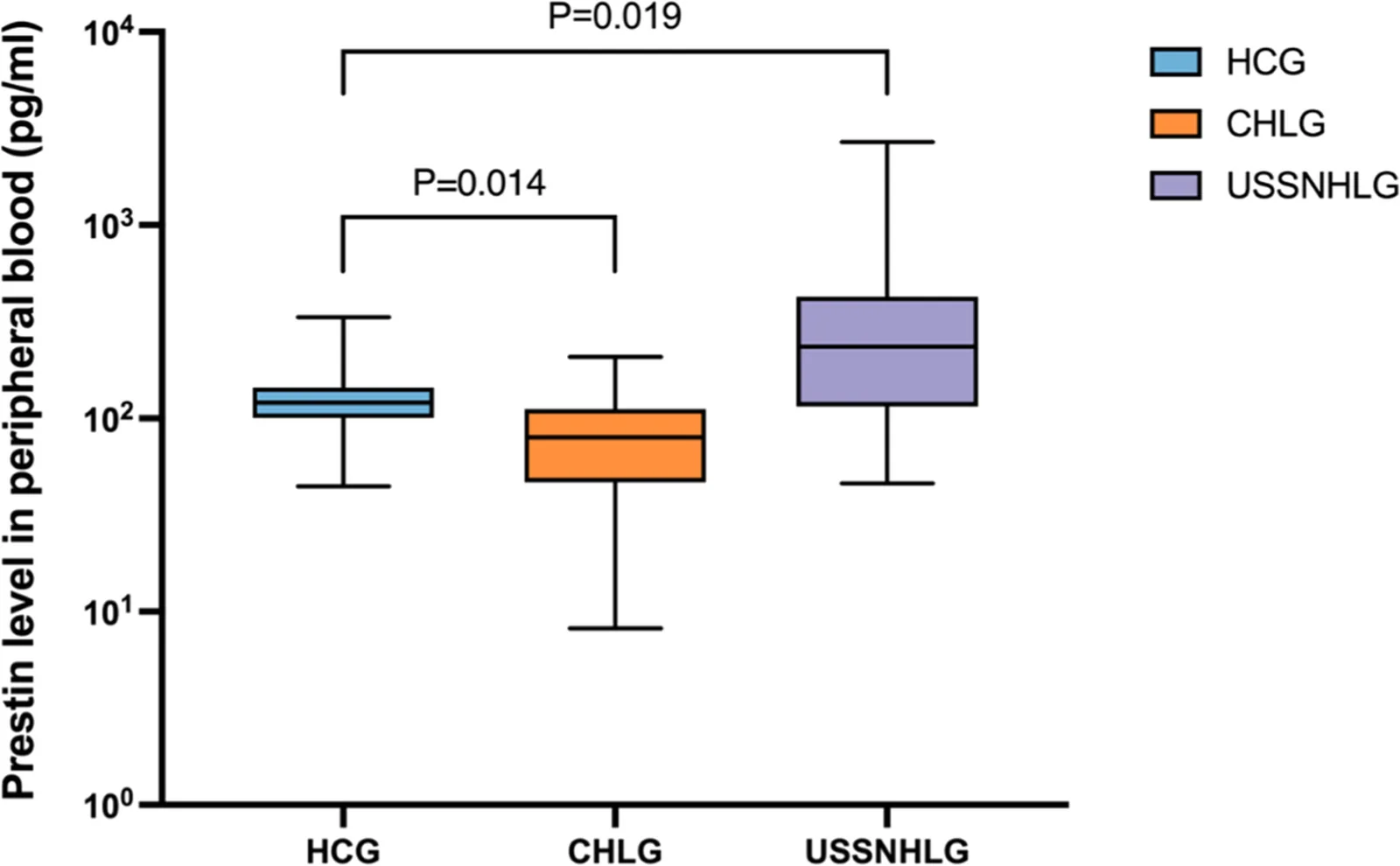
**Peripheral blood Prestin levels across the three study groups.** For optimal visualization of the data distribution, the y-axis representing peripheral blood Prestin concentrations (pg/mL) is presented on a base-10 logarithmic scale. Post hoc analyses were conducted using Dunn’s test with Bonferroni correction. Because only two biologically meaningful pairwise comparisons were predefined, the adjusted significance level was set at α’=0.025. The results demonstrated that Prestin levels in the HCG were significantly higher than those in the CHLG (*Z* = 2.46, p = 0.014) and significantly lower than those in the USSNHLG (*Z* = −2.34, p = 0.019). The pairwise comparison between the CHLG and USSNHLG was omitted, as it was not clinically relevant to the primary research questions of this study.

### Potential correlates of peripheral blood Prestin levels in USSNHL patients

To explore which factors correlate with Prestin levels in USSNHLG patients, Spearman correlation analysis was conducted between Prestin levels, age, and hearing status at various time points as quantitative variables. No correlations with clear clinical significance were newly identified (Fig. S1). However, no significant correlation was observed between baseline Prestin levels and post-treatment hearing thresholds (*R*=−0.220, p = 0.134).

The authors further evaluated associations between Prestin levels and Therapeutic outcomes or initial audiogram configurations. Table 2 presents the age and the pre-treatment average hearing threshold of the affected ear across different subgroups. Due to small sample sizes for upsloping (n = 3) and downsloping (n = 2) subtypes, subgroup analyses were restricted to flat (n = 21) and profound (n = 22) configurations (definitions provided earlier). There was no statistically significant difference in age among the subgroups.

**Table 2:** Comparison of baseline characteristics among patients with different therapeutic outcomes and audiogram curve types.

**Characteristic**	**Therapeutic Outcome Group**			**Audiogram Curve Group**	
	**Complete recovery** **(n=12)**	**Significant improvement** **(n=14)**	**No improvement** **(n=22)**	**Profound type** **(n=22)**	**Flat type** **(n=21)**
Age (years)	45.50 (33.75, 57.25)	59.00 (46.25, 64.50)	47.50 (36.75, 65.25)	47.50 (36.75, 61.75)	55.00 (45.00, 64.00)
Mean HT of AE (dB)	49 (32, 79)	79 (63, 106)	82 (60, 95)	93 (87, 111)	60 (47, 66)
Prestin (pg/mL)	419.33 (218.95, 488.00)	138.20 (80.41, 299.77)	230.36 (151.77, 379.07)	248.41 (110.56, 376.58)	193.39 (76.84, 456.07)

Mean HT of AE, Mean pre-treatment Hearing Threshold of Affected Ear.

Upon stratifying the patients into subgroups based on therapeutic outcomes, the median Prestin concentrations for the three subgroups were 419.33, 138.20, and 230.36 pg/mL, respectively. The Kruskal-Wallis *H*-test revealed no statistically significant differences in Prestin levels among these three subgroups (*H* = 4.56, df = 2, p = 0.102).

Similarly, when categorized by audiogram configurations, the median Prestin levels of the two resulting subgroups were 248.41 and 193.39 pg/mL, respectively. The Mann-Whitney *U test* indicated no significant difference between these two subgroups (*U* = 196.00, *Z* = −0.79, p = 0.430) (Fig. 2).

**Fig. 2:**
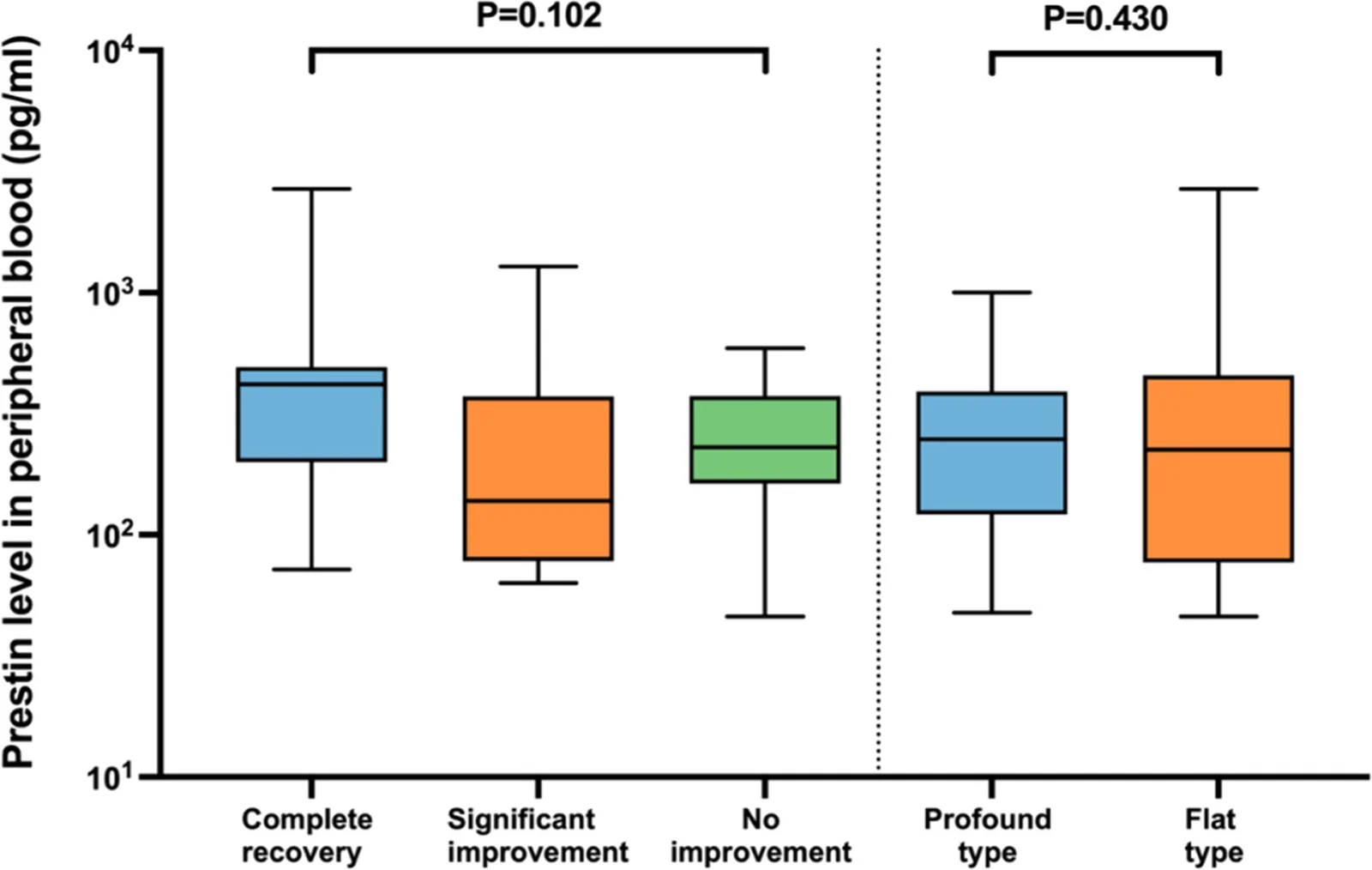
Correlation between Prestin levels and treatment outcomes or initial audiogram configurations. The x-axis represents subgroup classifications, and the y-axis indicates peripheral blood Prestin levels (pg/mL). Note: The y-axis uses a logarithmic scale with base 10.

## Discussion

### Theoretical basis for peripheral blood Prestin levels as an indicator of inner ear injury

Prestin, a motor protein localized to the lateral membranes of OHCs, is uniquely expressed in these cells.^11^ Consequently, serum Prestin levels are hypothesized to reflect OHC damage.^12^ Previous animal studies demonstrated that salicylate administration induces significant yet reversible increases in Prestin expression and levels in rat cochleae.^2^ Similarly, cisplatin-injected mice exhibited elevated serum Prestin levels from day 1 post-injection, persisting for 14 days, with greater sensitivity than Auditory Brainstem Response (ABR) thresholds.^3^ Short-term high-intensity noise exposure transiently increased serum Prestin levels in rats, but levels fell below baseline after 14 days as residual hair cells established a new equilibrium ‒ consistent with the present finding that peripheral blood Prestin levels may require over two weeks to stabilize post-acute injury.^13^ A scoping review by Iliadou et al.^14^ summarized that chronic high-level noise exposure may lead to reduced serum Prestin levels even in individuals with normal pure-tone thresholds, which was interpreted as a protective negative feedback mechanism.^14^ Current research on Prestin’s clinical utility in SSNHL largely agrees that its prognostic value is limited.^6^^,^^7^^,^^14^^,^^15^

Multiple factors may influence peripheral blood Prestin levels. First, a dynamic equilibrium likely exists between Prestin production and natural shedding from OHC lateral membranes, with shed Prestin entering the bloodstream via the Blood-Labyrinth Barrier (BLB). Second, hepatic and renal metabolism/excretion may modulate circulating Prestin levels. Under physiological conditions, structural constraints in OHC lateral membranes limit Prestin mobility.^16^ Acute inner ear injury disrupts this balance, increasing Prestin shedding and BLB permeability, thereby elevating peripheral levels. Chronic pathologies may establish a low-level equilibrium due to reduced acoustic stimulation.

### Interpretation of findings

Correlation between peripheral blood Prestin levels and hearing loss types

In this study, the peripheral blood Prestin levels in the HCG and CHLG were relatively low, with limited inter-individual variation. After numerical conversion, the values and degree of variation for peripheral blood Prestin in these three groups were close to those reported in previous studies by Parker et al.^17^ and Asli et al.^18^

As illustrated in Fig. 1, because the data did not meet the assumption of homogeneity of variances, the Kruskal-Wallis test was employed, revealing a highly significant overall difference among the three groups (p < 0.001). Post hoc analyses were subsequently conducted for two clinically relevant pairwise comparisons. To the best of our knowledge, this is the first study to report a significant reduction in peripheral blood Prestin levels in patients with chronic conductive hearing loss. This novel finding warrants further investigation to explore the inner ear alterations induced by persistently reduced acoustic input. Furthermore, the significant difference between the HCG and USSNHLG (p = 0.019, α’ = 0.025) is also noteworthy. This suggests that Prestin can serve as a valuable hematological biomarker reflecting acute inner ear damage.

Value of peripheral blood Prestin levels in USSNHL

In research exploring the application of Prestin as a biomarker in clinically relevant diseases, both Sun^6^ and Saadat^15^ have investigated peripheral blood Prestin levels in USSNHL, but their findings were inconsistent and limited by small sample sizes, leading to inconclusive results regarding the prognostic value of Prestin in USSNHL. Their studies were groundbreaking, yet the limited sample sizes hindered adequate subgroup analysis, leading to inconclusive results. In Figure S1 of this study, the authors performed Spearman correlation analysis between peripheral blood Prestin levels and age as well as hearing levels at different time points in 48 USSNHL patients. No significant correlation was found between Prestin levels and any of these indicators. Furthermore, within the USSNHLG group, Prestin levels among patients with different therapeutic outcomes showed a borderline overall significance (p = 0.102). However, the median levels of peripheral blood Prestin across the three groups displayed a counterintuitive trend (Complete recovery > No improvement > Significant improvement). Currently, no established theory or prior studies adequately explain this observation. Therefore, the present data do not support the utility of peripheral blood Prestin levels as a prognostic indicator for USSNHL.

### Limitations

Despite the preliminary insights provided by this exploratory study, several limitations must be acknowledged.

First, the sample size is relatively small, particularly within the USSNHL clinical subgroups. The limited number of cases for specific audiogram configurations (e.g., only 3 upsloping and 2 downsloping cases) restricted the authors’ ability to perform a comprehensive subgroup analysis. Furthermore, the small sample size may have limited the statistical power to detect significant differences in Prestin levels among patients with different therapeutic outcomes, potentially contributing to the borderline significance (p = 0.102) and the counterintuitive trend observed. Larger, multicenter cohorts are required to validate these findings.

Second, the measured Prestin concentrations may be influenced by the sampling and processing methods. Pre-analytical factors, such as the specific anticoagulants used (sodium citrate) and the centrifugation or storage conditions, might lead to an overall slight underestimation of the true circulating Prestin levels. Consequently, caution must be exercised when extrapolating the specific quantitative data, and these values should not yet be directly applied as universal clinical reference intervals.

Finally, a notable limitation of the current study design is the absence of a chronic Sensorineural Hearing Loss (SNHL) group. While our findings highlight the potential of peripheral Prestin as a biomarker for acute inner ear injury, its long-term dynamics remain unexplored. Given that chronic SNHL typically involves progressive and irreversible Outer Hair Cell (OHC) degeneration, Prestin release patterns in these patients may differ fundamentally from the acute surge observed in USSNHL. Consequently, the lack of a chronic SNHL cohort restricts the authors’ ability to fully characterize the dynamic equilibrium of Prestin across the entire spectrum of inner ear pathologies. Future studies incorporating patients with chronic SNHL are essential to comprehensively evaluate the temporal profile and diagnostic specificity of this biomarker.

## Conclusions

In conclusion, the present study confirms that peripheral blood Prestin levels are significantly elevated in patients with acute inner ear injury, supporting its potential as a biomarker for USSNHL. The authors also report a novel finding of reduced Prestin levels in patients with chronic conductive hearing loss, which warrants further investigation into the effects of reduced auditory input on Prestin dynamics. However, the present data do not support the use of Prestin as a prognostic biomarker for USSNHL. Future large-scale, multicenter studies with strict stratification by time from onset and hearing loss subtypes are needed to validate these findings.

## Authors’ contributions

Xuan Sun and Dian Wang: Conceptualization, data curation, formal analysis, and writing-original draft. Yahan Zhao and Zhenghan Zhu: Formal analysis; software. Lingxiang Hu and Yi Li: Resources, supervision, methodology, writing review & editing, and funding acquisition.

## Data availability statement

The data that support the findings of this study are not publicly available due to privacy reasons, but are available from the corresponding author, the first author, upon reasonable request.

## Statement of ethics

This study was performed in accordance with the Declaration of Helsinki. This human study was approved by the Ethics Committee of Beijing Tongren Hospital ‒ approval: TREC2024-KY023. All adult participants provided written informed consent to participate in this study.

## Funding

This study was supported by the following funding sources: Beijing High-level Innovation and Entrepreneurship Talent Support Program (G202521112) and Capital’s Funds for Health Improvement and Research (2024-1-1091).

## Conflicts of interest

The authors declare that there are no conflicts of interest relevant to this work, financial or otherwise.
