# Supplementary material for: Prestin as a potential biomarker in peripheral blood: differential expression across hearing loss subtypes and acute inner ear injury
Source: Clinics (Sao Paulo). 2026 Oct 15;81:101143. doi: 10.1016/j.clinsp.2026.101143 (PMC13691484; doi:10.1016/j.clinsp.2026.101143)
Supplement: Supplementary file 1 [file mmc1.docx]

CLINICS-D-25-01826_Supplementary Material

**Figure S1** Spearman Correlation Analysis between Prestin and Selected Quantitative Variables. The upper number in each box in the upper-right section is the correlation coefficient, and the lower number is the corresponding p-value. The lower-left section shows the corresponding scatter plots and fitted lines. HTI-AE, Hearing Threshold Improvement in Affected Ear; MPreTx HT (AE), Mean Pre-treatment Hearing Threshold of Affected Ear; MPostTx HT (AE), Mean Post-treatment Hearing Threshold of Affected Ear; MPreTx HT (BE), Mean Pre-treatment Hearing Threshold of Both Ears.
